# Prescribing patterns of antibiotics according to the WHO access, watch, and reserve (AWaRe) classification in the pediatric outpatient department: A prospective study

**DOI:** 10.12669/pjms.41.11.12169

**Published:** 2025-11

**Authors:** Sobia Ramzan, Afia Tariq Butt, Waqar Khowaja, Siddiqa Ghani

**Affiliations:** 1 Sobia Ramzan, (SR), FCPS - Pediatrics, MRCPCH. Senior Instructor, Department of Pediatric Medicine and Child Health, Aga Khan University Hospital, Karachi, Pakistan; 2 Afia Tariq Butt, (ATB), FCPS - Pediatrics, MRCPCH. Senior Instructor, Department of Pediatric Medicine and Child Health, Aga Khan University Hospital, Karachi, Pakistan; 3 Waqar Khowaja, (WK), FCPS - Pediatrics, FCPS – Neonatology. Clinical Assistant Professor, Department of Pediatric Medicine and Child Health, Aga Khan University Hospital, Karachi, Pakistan; 4 Siddiqa Ghani, (SG), FCPS – Pediatrics. Senior Instructor, Department of Pediatric Medicine and Child Health, Aga Khan University Hospital, Karachi, Pakistan

**Keywords:** Antibiotics, AWaRe classification, Antimicrobial resistance, Outpatient care, Pediatric prescribing

## Abstract

**Objectives:**

Antimicrobial resistance (AMR) poses a significant global health threat, disproportionately affecting young children, especially in low- and middle-income countries. The World Health Organization (WHO) introduced the Access, Watch, and Reserve (AWaRe) classification system to guide responsible antibiotic prescribing. This study evaluates antibiotic prescribing patterns in pediatric outpatient departments in Pakistan using the WHO AWaRe framework.

**Methodology:**

A prospective, cross-sectional, multicentric study was conducted in four urban Level two teaching hospitals affiliated with Aga Khan University Hospital in Pakistan. Data was collected monthly for one year (August, 2023 - July, 2024), involving pediatric patients aged one month to twelve years. Prescriptions were categorized into AWaRe groups, with frequencies analyzed by clinical indication and patient age.

**Results:**

Of 1,802 pediatric encounters analyzed, antibiotics were prescribed in 529 cases (29.4%), predominantly orally (77.9%). Azithromycin (19.5%) and Amoxicillin-Clavulanic acid (19.1%) were the most commonly prescribed agents. Watch antibiotics represented 69.2% of prescriptions, significantly exceeding WHO recommendations (≤40%). Antibiotic prescription rates varied substantially by diagnosis, being highest for enteric fever (89.6%), sepsis (82.4%), and UTIs (75.0%), with notable inappropriate prescribing for viral conditions such as URTIs (33.2%), acute febrile illness (31.7%), and bronchiolitis (31%). Narrow-spectrum Access antibiotics were underutilized (29.7%), while Reserve antibiotics were rarely prescribed (1.1%).

**Conclusion:**

This study concludes that the poor implementation of AWaRe among pediatricians in Pakistan indicates urgent need for multiple interventions. There is a dire need to improve antibiotic prescribing practices, ranging from antibiotic prescription policies, training of pediatricians in antibiotic use, AWaRe implementation, and regular monitoring in the form of clinical audits.

## INTRODUCTION

Antimicrobial resistance (AMR) is a major global health challenge, reducing the effectiveness of treatments and increasing mortality. In 2019, drug-resistant infections contributed to nearly five million deaths, with 1.26 million directly attributed to resistance.^1^ Children under five are disproportionately affected by increasing AMR, accounting for 20% of AMR-related deaths in 2019.^2^ The highest burden falls on low- and middle-income countries (LMICs), especially South Asia.^1^ WHO estimates report around 389,000 annual deaths attributed to AMR in South Asia.^3^ India, Pakistan, and Bangladesh face worsening trends, with projections estimating over two million AMR-related deaths annually in India by 2050.^4^

To tackle AMR, the World Health Organization (WHO) introduced the Global Action Plan, focusing on surveillance and responsible antimicrobial use.^5^ A central component is the Access, Watch, and Reserve (AWaRe) framework, which categorizes antibiotics to guide prescribing and monitoring.^6^,^7^ Access’ includes first-line agents with lower resistance risk; ’Watch’ covers broad-spectrum drugs needing cautious use; and ’Reserve’ is for last-resort options against multidrug-resistant infections. WHO targets at least 60% of antibiotic use within the Access group to mitigate resistance.^7^ The AWaRe framework is especially valuable in high-AMR regions with limited surveillance, like Pakistan, where it aids antimicrobial stewardship by promoting narrow-spectrum antibiotics.^8^ The country’s National Action Plan supports this approach through AMR awareness, monitoring, infection control, optimized prescribing, and sustainable policies.^8^

Despite the urgency, most research on antibiotic use focuses on adults, leaving a gap in pediatric prescribing data, particularly in LMICs like Pakistan.^9^,^10^ This study addresses that gap by analyzing pediatric outpatient antibiotic prescribing patterns at secondary care centers using the AWaRe classification. In 2021, the WHO Essential Medicines List for Children adopted AWaRe with a traffic light system-green for Access, amber for Watch, and red for Reserve.^6^ The findings of this study aim to improve pediatric prescribing practices and support stewardship efforts to combat AMR in vulnerable populations.

## METHODOLOGY

A prospective, cross-sectional, multicenter study was conducted across four secondary health care facilities located in Garden, Karimabad, Kharadar, and Hyderabad. These hospitals have OPDs, nurseries, well-baby units, pediatric wards, and urgent care units but lack neonatal and pediatric intensive care. All these four facilities are affiliated with Aga Khan University Hospital (AKUH), Karachi. Data were collected prospectively from pediatric OPDs of all the selected hospitals.

### Inclusion and Exclusion Criteria:

The study included pediatric patients aged between one month and twelve years who visited pediatric OPDs on the 15th day of the month. The data was collected each month from August 2023 to July 2024. Exclusions included encounters without a diagnosis or medication prescription, incomplete patient data, hospital admissions from OPDs, or post-discharge follow-ups. Patients prescribed topical antimicrobials, antifungals, antivirals, anthelmintics, antiprotozoals, or antituberculosis medications were also excluded from the study, as these medications are not included in the WHO AWaRe classification.

### Ethical consideration:

This study was exempted from routine ethical considerations by the Institutional Review Board (IRB) of Aga Khan University Hospital and was assigned the No: 2023-8930-25959; dated: Aug. 3, 2025.

### Operational Definitions:

An ”encounter” refers to a single consultation in one department, while a ”prescription” includes all drugs prescribed during a single encounter. ”Antibiotics” in this study refer to antibacterial agents for systemic use classified under the Anatomical Therapeutic Chemical (ATC) class J01, along with orally administered metronidazole (P01AB01).

### Sample size:

A random day was selected to collect data of all the eligible children presenting on that day, i.e. 15^th^ day of each month. Hence, all children aged one month to twelve years who visited the OPD on the 15th day of the month between 15^th^ August 2023 and 15^th^ July 2024 were included, provided they met the inclusion criteria.

### Data collection and variables:

Prescription records were collected from the hospital information system (HIMS) on the fifteenth of each month for one year, and outpatient files from each level two hospital were reviewed within a week. Data included the total number of pediatric OPD visits, systemic antibiotic prescriptions, and patient details such as age, gender, diagnosis, prescribed antibiotics, and the route of administration (IV/oral).

### Statistical analysis:

Data were entered on Microsoft Excel and later exported to SPSS for analysis (version 28.0, SPSS, IBM, and Armonk, New York, USA). Descriptive analysis was performed with variables being presented as frequencies and percentages.

Antibiotic prescription indicators analyzed included the overall prescription rate, use of intravenous antibiotics, combination therapy, percentage distribution of antibiotic classes (i.e., Third generation Cephalosporins, Macrolides, Beta-lactamase-inhibitors, Fluoroquinolones, Penicillins, Imidazoles, Carbapenems, First generation Cephalosporins, Second generation Cephalosporins), and AWaRe classification. These indicators were evaluated separately for each diagnostic condition (as diagnosed by the physician on the basis of clinical information, including relevant investigations) and age group, categorized as: zero to three months, three months to two years, two to six years, and six to twelve years.

### Outcomes:

The study aimed to determine the frequency of prescribed antibiotics classified under Access, Watch, and Reserve groups in the pediatric OPDs of Level two care centers of AKUH according to WHO AWaRe classification over a one-year period (August 2023-July 2024). The frequency of antibiotic prescriptions was analyzed by age group and clinical indication.

## RESULTS

A total of 1,802 pediatric outpatient encounters were analyzed. In 529 cases (29.4%), antibiotics were prescribed, which included oral antibiotics in 412 patients (22.9%) and intravenous (IV) antibiotics in 117 patients (6.5%) (Table-I).

**Appendix: Table I:** Breakdown of encounters.

Variable	Value
Total number of encounters	1802
Total number of encounters in which antibiotics were prescribed (n, %)	529 (29.4)
Encounters in which oral antibiotics were prescribed (n, %)	412 (22.9)
Encounters in which IV antibiotics were prescribed (n, %)	117 (6.5)

Azithromycin (19.5%) and Amoxicillin-Clavulanic acid (19.1%) were the most frequently prescribed antibiotic, followed by Cefixime (18.7%). The major classes were third-generation cephalosporin (35.8%), macrolides (25%), and beta-lactamase inhibitors (19.1%), suggesting that these broad-spectrum agents are preferred. Fluoroquinolones were 9.5% and the other classes, imidazole (1.1%) and carbapenems (0.9%), were infrequently used (Table-II).

**Table-II:** Breakdown of frequency of antibiotic agents and classes.

Agents/Classes	Frequency (%)
***Antibiotic agents (top 10)***	
Azithromycin	103 (19.5)
Amoxicillin + Clavulanic acid	101 (19.1)
Cefixime	99 (18.7)
Ceftriaxone	50 (9.5)
Ciprofloxacin	50 (9.5)
Cefpodoxime	31 (5.9)
Clarithromycin	29 (5.5)
Amoxicillin	21 (4.0)
Ampicillin	11 (2.1)
Cefotaxime	9 (1.7)
Entamizole	5 (0.9)
Meropenem	5 (0.9)
***Antibiotic classes (top 10)***	
Third-generation Cephalosporins	189 (35.8)
Macrolides	132 (25.0)
Beta-lactamase-inhibitors	101 (19.1)
Fluoroquinolones	50 (9.5)
Penicillins	32 (6.1)
Imidazoles	6 (1.1)
Carbapenems	5 (0.9)
First-generation Cephalosporins, Second-generation Cephalosporins	3 (0.6)
Aminoglycosides, Aminoquinolene, Glycopeptides, Lincosamides, Oxazolidinones, Phosphonics, Rifamycin	1 (0.2)

Antibiotic prescribing varied by diagnosis, with the highest rates in enteric fever (89.6%), sepsis (82.4%), and UTIs (75.0%). IV antibiotics were commonly used for enteric fever (41.7%) and sepsis (64.7%), while UTIs were primarily treated orally (62.5%). Pneumonia cases also had significant antibiotic use (66.7%). Notable percentage are also prescribed for viral conditions, including URTIs (33.2%), acute febrile illness (31.7%), and bronchiolitis (31%), highlighting their overuse in OPDs (Table-III).

**Table III:** Breakdown of mode of delivery of antibiotics relative to diagnosis.

Diagnosis	Number of encounters	Encounters in which antibiotics were prescribed (%)	Encounters with oral antibiotics prescribed (%)	Encounters with IV antibiotics prescribed (%)
Enteric fever	48	43 (89.6)	23 (47.9)	20 (41.7)
Sepsis	17	14 (82.4)	3 (17.6)	11 (64.7)
UTI	16	12 (75.0)	10 (62.5)	2 (12.5)
Pneumonia	159	106 (66.7)	74 (46.5)	32 (20.1)
Diarrhea	205	79 (38.5)	58 (28.3)	21 (10.2)
URTI	648	215 (33.2)	197 (30.4)	18 (2.8)
Acute febrile illness	63	20 (31.7)	16 (25.4)	4 (6.3)
Bronchiolitis	42	13 (31.0)	9 (21.4)	4 (9.5)
Surgical problems	23	5 (21.7)	2 (8.7)	3 (13.0)
Skin infection	44	8 (18.2)	7 (15.9)	1 (2.3)
Viral exanthem	15	2 (13.3)	2 (13.3)	0
Gastritis	84	6 (7.1)	5 (6.0)	1 (1.2)
Seizure disorder	20	1 (5.0)	1 (5.0)	0
Developmental delay	23	1 (4.3)	1 (4.3)	0
Malnutrition	75	3 (4.0)	3 (4.0)	0
Routine checkup	254	1 (0.4)	1 (0.4)	0

Disease patterns showed different patterns through different childhood age groups. URTIs were the most common diagnosis in every age range, but children between three months and two years had the most (51.3%) URTI diagnoses. Diarrhea and bronchiolitis were also prevalent (62.9% and 62.2% of cases, respectively) in this age group, respectively, followed by pneumonia, which occurred in 54.5% of cases. Sepsis was more common in neonates (zero to three months), accounting for 75% of the cases. However, enteric fever was predominantly found in children more than two years of age (Table-IV).

**Table-IV:** Categorizing diagnosis relative to age groups.

Diagnosis	0-3 months	3 months - 2 years	2 years - 6 years	6 years - 12 years
URTI	83 (13.1)	326 (51.3)	153 (24.1)	74 (11.6)
Routine checkup	146 (59.3)	67 (27.2)	23 (9.3)	10 (4.1)
Diarrhea	12 (5.9)	129 (62.9)	36 (17.6)	28 (13.7)
Pneumonia	11 (7.1)	84 (54.5)	39 (25.3)	20 (13.0)
Gastritis	18 (21.4)	21 (25.0)	20 (23.8)	25 (29.8)
Malnutrition	4 (5.3)	31 (41.3)	18 (24.0)	22 (29.3)
Acute febrile illness	1 (1.6)	25 (40.3)	24 (38.7)	12 (19.4)
Enteric fever	1 (2.1)	12 (25.0)	19 (39.6)	16 (33.3)
Skin infection	13 (29.5)	21 (47.4)	4 (9.1)	6 (13.6)
Bronchiolitis	14 (37.8)	23 (62.2)	0	0
Constipation	4 (14.3)	13 (46.4)	8 (26.8)	3 (10.7)
Surgical problems	4 (17.4)	5 (21.7)	8 (34.8)	6 (26.1)
Developmental delay	2 (8.7)	7 (30.4)	8 (34.8)	6 (26.1)
Seizure disorder	0	6 (30.0)	6 (30.0)	8 (40.0)
Colic	15 (88.2)	2 (11.8)	0	0
Sepsis	12 (75.0)	0	2 (12.5)	2 (12.5)
UTI	0	7 (43.8)	5 (31.3)	4 (25.0)

Antibiotic use varied by age. Azithromycin (54.5%), Cefixime (46.9%), Ciprofloxacin (68%), and Cefpodoxime (72%) were commonly prescribed for children aged three months to two years. Amoxicillin + Clavulanic Acid was frequently given to children aged two to six years (39.6%). In neonates (zero to three months), Ampicillin and Cefotaxime were predominantly used (88.9%) due to their higher susceptibility to systemic infections (Table-V).

**Table-V:** Categorizing antibiotic agents relative to age groups.

Antibiotics	0-3 months	3 months - 2 years	2 years - 6 years	6 years - 12 years
Azithromycin	0 (0.0)	55 (54.5)	30 (29.7)	16 (15.8)
Amoxicillin + Clavulanic acid	6 (5.9)	38 (37.6)	40 (39.6)	17 (16.8)
Cefixime	4 (4.1)	46 (46.9)	26 (26.5)	22 (22.4)
Ceftriaxone	2 (4.3)	26 (55.3)	9 (19.1)	10 (21.3)
Ciprofloxacin	0	34 (68.0)	9 (18.0)	7 (14.0)
Cefpodoxime	1 (3.2)	22 (71.0)	4 (12.9)	4 (12.9)
Clarithromycin	2 (7.1)	17 (60.7)	5 (17.9)	4 (14.3)
Amoxicillin	7 (33.3)	10 (47.6)	4 (19.0)	0
Ampicillin	8 (88.9)	1 (11.1)	0	0
Cefotaxime	8 (88.9)	1 (11.1)	0	0
Entamizole	0	2 (40.0)	2 (40.0)	1 (20.0)
Meropenem	0	0	4 (80.0)	1 (20.0)
Cefaclor	1 (33.3)	2 (66.7)	0	0
Cephalexin	2 (100)	0	0	0
Metronidazole	0	2 (100)	0	0
Amikacin	0	0	1 (100)	0

**Fig.1:**
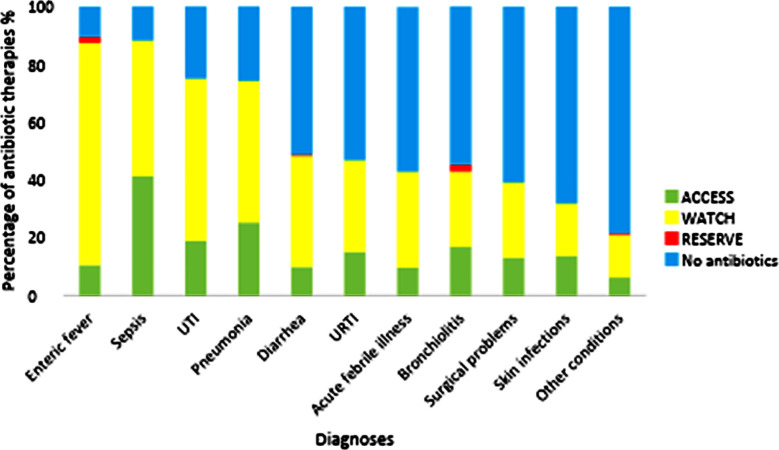
Access, Watch, and Reserve with Diagnosis.

The Watch group, comprising broad-spectrum antibiotics, were used more frequently than Access antibiotics, with AWaRe classification showing Access (29.7%), Watch (69.2%), and Reserve (1.1%) usage. Watch antibiotics were primarily prescribed for enteric fever (75%), UTIs (55%), pneumonia (50%), diarrhea and URTIs (40% each), acute febrile illness (35%), and bronchiolitis (30%), indicating overuse in pediatric OPDs. In contrast, narrow-spectrum Access antibiotics, recommended as first-line treatment, were mainly used for sepsis (40%) and less frequently for other conditions, reflecting under-reliance on these agents for common pediatric infections. Reserve antibiotics, restricted for multidrug-resistant infections, were minimally prescribed across all diseases, aligning with stewardship practices by being reserved for severe or resistant cases (Fig.1).

**Fig.2:**
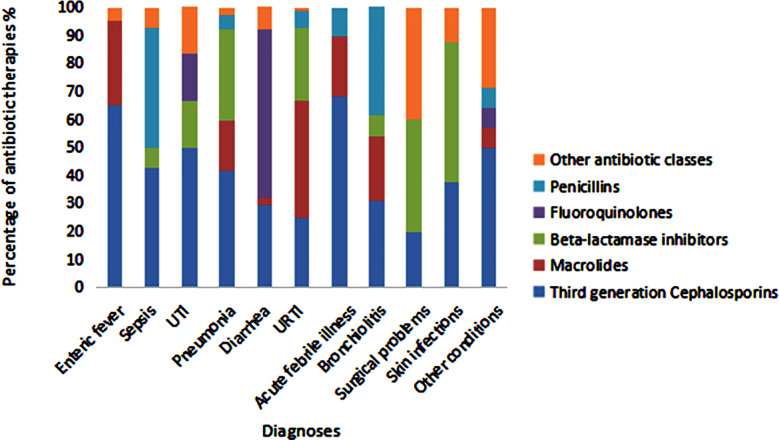
Antibiotic Distribution Across Various Diagnoses.

Third-generation cephalosporins are mainly prescribed for acute febrile illness, enteric fever, UTIs, and pneumonia, while Macrolides are common for URTIs, enteric fever, and bronchiolitis. Beta-lactamase inhibitors are frequently used for skin infections, surgical cases, pneumonia, and URTIs, whereas Penicillins are preferred for sepsis and bronchiolitis. Fluoroquinolones, though less used overall, are most prescribed for diarrhea and UTIs. The data reflect a preference for broad-spectrum antibiotics despite some adherence to narrow-spectrum options like Penicillins and Beta-lactamase inhibitors (Fig.2).

## DISCUSSION

This study found a 29.4% prevalence of antibiotic prescriptions in pediatric outpatient departments (OPDs) of secondary care centers, highlighting significant reliance on antibiotics. Although lower than inpatient rates, this underscores the need for ongoing evaluation and stronger antimicrobial stewardship. Most prescriptions (77.9%) were oral, reflecting outpatient preferences for non-invasive treatment, while 22.1% involved IV antibiotics, typically reserved for severe infections requiring rapid action.^13^ Our study found a lower proportion of Access antibiotics (29.7%) than the WHO-recommended 60% target.

The high use of Watch antibiotics (69.2%) aligns with recent evidence from Pakistan.^11^-^14^ A study conducted in 2024 among pediatric in-patients secondary care centers in Karachi showed watch antibiotics use exceeded 80% emphasizing the critical need for enhanced antimicrobial stewardship.^15^ Similarly, pediatric studies in China, report that 82.2% of antibiotic prescriptions indicate broad-spectrum antibiotic overuse.^16^ A global study across 76 countries also reported a 90.9% rise in Watch antibiotic use over 15 years, particularly in LMICs.^17^

The current study found third-generation cephalosporins (35.8%), particularly cefixime (18.7%) and ceftriaxone (9.5%), were the most prescribed antibiotics, followed by macrolides (25%), mainly azithromycin (19.5%), and beta-lactamase inhibitors (19.1%). These findings are well supported by recent evidence from Pakistan.^18^,^19^

The antibiotic use in current study also aligns with global trends of excessive Watch group antibiotic use in pediatric outpatient care. A nationwide study in China (2017-2019) reported high third-generation cephalosporin use (17.4%), with Watch antibiotics comprising 55.0% of prescriptions.^20^,^21^ In outpatient settings, despite guidelines favoring amoxicillin, in Ethiopia, ceftriaxone was frequently prescribed inappropriately.^22^ The frequent use of broad-spectrum Watch group antibiotics over narrow-spectrum Access antibiotics is a concerning trend, emphasizing the need to reinforce the WHO AWaRe framework guidelines in outpatient settings.

In this study prescribing trends varied by clinical indication. Antibiotic use was highest in enteric fever (89.6%), sepsis (82.4%), and urinary tract infections (75%), as expected for bacterial infections. However, substantial use in pneumonia (66.7%), diarrhea (38.5%), acute febrile illness (31.7%), bronchiolitis (31%), and URTI (33.2%) raises concerns about over prescription for conditions often of viral origin. A recent study in Pakistan found that 39% of hospitalized children with acute watery diarrhea received unnecessary antibiotics despite guidelines recommending fluid-based management.^23^ A multicenter outpatient study in China reported antibiotic use in 41% of pediatric URTIs, despite their viral nature. The same study also found frequent broad-spectrum antibiotic use for acute febrile illness without confirmed bacterial infection.^24^ Similarly, an outpatient study in Tennessee showed that 31% of bronchiolitis cases were treated with antibiotics despite guidelines recommending supportive care.^25^

These findings reflect a universal challenge in antibiotic prescribing patterns, emphasizing the need for stronger antimicrobial stewardship efforts globally, particularly in resource-limited settings like Pakistan. Regional variations in antibiotic prescribing reflect differences in healthcare infrastructure and local practices. In Pakistan, insufficient knowledge about antibiotic use and AMR among pediatricians is the main reason behind irrational use of antibiotics among children.^26^ A study in Bangladesh linked high prescription rates to socioeconomic constraints and weak stewardship, while research in Latin America attributed them to healthcare accessibility and guideline differences.^27^,^28^ These discrepancies underscore the need for country-specific interventions tailored to local healthcare systems and resources.

This multicenter study provides very first evidence from Pakistan regarding local patterns of antibiotic prescribing in pediatric outpatient settings. It is also the first prospective, outpatients based, multicenter study in an LMIC to apply the WHO AWaRe classification at secondary care hospitals.

Hence, this study provides valuable evidence from multiple secondary care centers in Karachi, highlighting substantial overuse of broad-spectrum Watch antibiotics and underuse of Access antibiotics, even for viral conditions. This study indicates substantial gaps in the knowledge of pediatricians and suggests further research to study the current status of pediatricians’ knowledge regarding antibiotic use and relevant training.

### Strengths and limitations:

Key strengths of our study are the prospective design, multicenter coverage, and use of the WHO AWaRe framework, enhancing data robustness and global comparability. The detailed stratification by age and diagnosis offers practical insights for stewardship programs in pediatric outpatient care.

### Limitations:

It includes the lack of microbiological data, which restricts the assessment of prescription appropriateness. Data collection on one day per month limits seasonal analysis. Urban-only settings also reduce the applicability of study findings to rural or primary care environments. Future studies should assess clinical competency for antibiotic use, appropriateness using culture data, explore prescriber rationale, and evaluate interventions such as digital decision tools and audits. Expanding research to rural and primary care will support more comprehensive, scalable antibiotic stewardship strategies.

## CONCLUSION

This study concludes that the poor implementation of AWaRe among pediatricians in Pakistan indicates urgent need for multiple interventions. There is a dire need to improve antibiotic prescribing practices, ranging from antibiotic prescription policies, training of pediatricians in antibiotic use, AWaRe implementation, and regular monitoring in the form of clinical audits.

### Author’s Contribution:

**SR:** Conceptualization, study design, interpretation of data, manuscript writing, and final revision.

**ATB:** Data collection, statistical analysis, and manuscript writing.

**WK and SG:** Data collection. Critical Review.

**SR and ATB:** Take full responsibility for the accuracy and integrity of the work.

All authors have read and approved the final version of the manuscript.
